# Biphasic human insulin 30 thrice daily, is it reasonable?

**DOI:** 10.1186/s13104-020-05090-6

**Published:** 2020-05-24

**Authors:** Nesreen A. Saadeh, Ola Y. Al-Azzeh, Yousef S. Khader

**Affiliations:** 1 Department of Internal Medicine, Faculty of Medicine, Jordan University of Science and Technology, P.O.Box 3030, Irbid, 22110 Jordan; 2 Pharmacy Practice Department, College of Pharmacy, King Saud Bin Abdulaziz University for Health Sciences, Riyadh, Saudi Arabia; 3 Department of Community Medicine, Public Health and Family Medicine, Faculty of Medicine, Jordan University of Science & Technology, Irbid, Jordan

**Keywords:** Biphasic human insulin 30, Thrice daily insulin injections, Twice daily insulin injections

## Abstract

**Objective:**

To evaluate the efficacy and safety of thrice daily Biphasic Human Insulin 30 (BHI 30) versus the traditional twice-daily regimen in type 2 diabetes mellitus (T2DM) patients. It’s a cross over single clinical study. Twenty-two diabetic patients who were already using BHI 30 in twice or thrice daily regimens with or without metformin were included. At the 1st interval; patients continued on their usual insulin regimen as twice or thrice daily injections with adjustment of insulin doses guided by their glucose readings. On the 2nd interval; patients were switched to the other regimen with the same total daily insulin dose redistributed.

**Results:**

There was a significant decrease in HbA1c level (p < 0.05) at the end of the first 3 months of trial regardless on which regimen the patient started, but there was no significant difference in the mean HbA1c reduction in patients when they were on twice daily insulin injections (1.1 ± 1.3) versus the time they were on thrice daily insulin injections (0.8 ± 1.71), p > 0.05. On the other hand, patients had lower average blood glucose readings (mg/dl) when they were on thrice daily insulin injections (161.4 ± 62.7) compared to twice daily regimen (166.0 ± 69.5), p < 0.05.

## Introduction

Several studies, most prominently the Diabetes Control and Complications Trial (DCCT) and the United Kingdom Prospective Diabetes Study, have confirmed that more intensive management of blood glucose control reduces the incidence and delays the progression of late diabetic complications associated with Type 1 and Type 2 diabetes [1, 2].

Insulin is used to control patients with diabetes, not controlled on oral hypoglycemic agents aiming for a more intensive glycemic control. Basal bolus insulin analogue regimens are regarded as the best way to achieve that goal, but the cost of the new analogues and the multiple daily injections make their use less likely than the conventional regimens using pre-mixed insulin twice daily.

Experience has shown that twice daily treatment with the most widely used mixture of 30% soluble human insulin (HI) and 70% protamine-crystallized human insulin (NPH); Biphasic Human Insulin 30 (BHI 30) partially may compensate for hyperglycemia after breakfast and dinner, as well as provide sufficient basal insulin requirements until the next injection [3].

Trials have been conducted to prove efficacy of regimens using pre-mixed insulin analogues in a thrice daily injection regimen to achieve a better glycemic control, but a few studies have experimented with the traditional BHI 30. Clements et al. concluded that thrice daily biphasic insulin Aspart (BIAsp) can safely be used to intensify treatment for patients inadequately controlled on twice-daily BHI 30 [4]. Also, Yang et al. showed that thrice daily BIAsp 30 offered a greater reduction in A1C without increasing risk of hypoglycemia, insulin dose, and weight gain, especially in subjects with A1C > 9% (75 mmol/mol) [5].

Another study has shown that thrice daily biphasic human insulin regimen is non-inferior to the basal- bolus insulin analogue regimen in terms of efficacy and safety in patients with poorly controlled type 2 diabetes mellitus [6].

The insulin widely used in our country is BHI 30, since it is cheap and covered by all medical insurance companies. A widely used practice is intensifying insulin regimen using BHI 30 in a thrice daily fashion.

The objective of the present study was to compare the efficacy and safety profile of biphasic human insulin 30 (BHI 30) when used twice daily as opposed to thrice daily injection regimen.

## Main text

### Methods

#### Study design

This trial was designed as an observational, open-label, single-center crossover study. It was carried out at the diabetes and endocrinology clinic at King Abdullah University Hospital (KAUH) in May 2014.

**Fig. 1:**
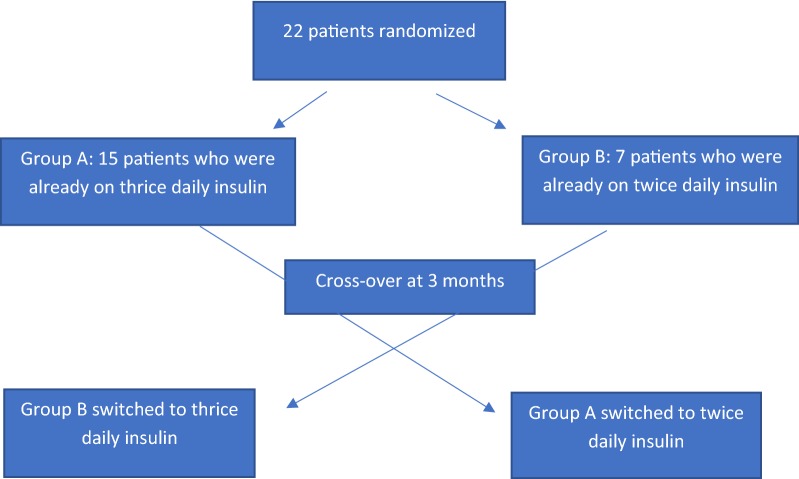
Patient distribution

Patients were assigned into two groups based on their insulin regimen (Fig. 1). Group A were patients already on thrice daily insulin regimen and group B were patients already on twice daily insulin regimen. They were followed for 3 months. After that, patients in group A were switched from thrice daily insulin regimen to twice daily insulin regimen, and patients of group B were switched from twice daily insulin regimen to thrice daily insulin regimen. Then they were followed for another 3 months. Therefore, the total study period lasted for 6 months. During each 3–month interval, patients were advised to comply with each interval recommendations (i.e. insulin regimen). However, adjustments to insulin doses were done based on glucose readings.

The design of this study, including clinical and laboratory measurements, was approved by Jordan University of Science and Technology Institutional Review Board (IRB) committee and was carried out in accordance with the Helsinki Declaration of 1975, with all amendments and revisions.

#### Study sample

A total of 22 unrelated diabetic patients completed the study protocol. To compute the required sample size, G power software version 3.1 was used. At a power of 80%, effect size equal to 0.5 and alpha error of 0.05, 27 patients were computed as the required sample size. Fifty people who met the inclusion criteria were invited to participate, but only 22 were able to complete the 6-month period of the study, for which their data was used in the analysis.

Inclusion criteria consisted of Type 2 diabetic patients who were already on the conventional Biphasic Human Insulin 30 (BHI 30). However, researchers excluded patients who were taking any oral antidiabetic agents other than Metformin. Also, patients with an active neoplasm or a history of neoplasm, severe liver dysfunction, severe renal failure, major surgery within 2 weeks of enrolment, alcohol abuse, critically ill patients, patients with a significant history of hypoglycemic comas and patients on steroids were excluded.

#### Study measurements

Before patients were recruited, they were offered to participate in the study provided that they had to change their regimen after 3 months, including adjustment of doses. Patients who agreed to participate and met the inclusion criteria signed a consent form. At the time of recruitment, patients were informed about the objectives of the study, study protocol and duration of the study. Afterwards, educational sessions were conducted, which included a brief review on diabetes mellitus, lifestyle modifications, symptoms of hypo- and hyperglycemia, treatment options, complications, comprehensive target glycemic goals and proper glucose monitoring using glucometers, and insulin adjustment techniques guided by their self-monitoring of blood glucose (SMBG) readings. To ensure a standard SMBG reading by all patients, a reading demonstration was done on a standard Accu-Chek Performa^®^ glucometer 2 times. Each patient was given the Accu-Chek Performa^®^ glucometer and was asked to use for SMBG readings at home as demonstrated.

The initial visit included collection of demographic information and medical history in addition to body mass index (BMI) measurements.

Laboratory measurements at the initial visit included tests of FBS, HbA1c, thyroid function test, lipid profile, kidney function test, liver function test, urinalysis, spot microalbuminuria test, and complete blood count. Those measurements were repeated at the end of each 3-month interval.

For self-measurement of glucose at home, patients were asked to report 4–6 readings/day based on the number of daily meals. These readings were fasting, pre-meals, 2-h postprandial, in addition to any time of feeling hypoglycemic. Special diaries were distributed for the patients in order to document their readings along with any hypoglycemic episodes.

For the rest of the visits, during the 3-month interval, visits to the clinic were scheduled after 1 week, 4 weeks, 8 weeks, and 12 weeks. On each of these visits, home readings of capillary blood glucose were collected. To validate home readings, the hard copy documented by patients was compared to a soft copy extracted from the ACCU-CHEK Smart Pix Device Reader.

At the end of 3 months (12 weeks), patients of each group were switched to the other regimen (i.e. Twice Daily BHI 30 to Thrice Daily BHI 30; and vice versa). All measurements done at the initial visit and the follow-up visits were repeated as above.

#### Statistical analysis

Data were described and analyzed using the IBM SPSS Statistics (version 20). Data were described using means, standard deviations, or percentages wherever appropriate. The means of the studied parameters at the baseline and after 3 months were compared using paired t test. The differences in the change (Baseline—after 3 months) of the studied parameters including HBA1c between the two treatment regimens were tested using independent t test. A *p* value of less than 0.05 was considered statistically significant.

### Results

A total of 22 patients completed the study protocol, of whom 68% were females. The mean age of patients was 55.0 (± 10.7) years. Table 1 illustrates the demographic information of participants. A significant decrease in HbA1c level (*p *< 0.05) was observed at the end of the first 3 months of trial regardless on which regimen the patient started, but there was no significant difference in the mean HbA1c reduction in patients when they were on twice daily insulin injections versus the time they were on thrice daily insulin injection (1.1 ± 1.3 vs. 0.8 ± 1.71, respectively, *p *> 0.05).

**Table 1:** Patient demographics

Baseline characteristics	All participants
Mean age (years), mean (SD)	55.0 (10.7)
Female, n (%)	15 (68.2)
Education > high school, n (%)	5 (36.3)
Married, n (%)	21 (95.4)
Mean BMI (kg/m^2^), mean (SD)	32.7 (4.3)
Baseline HbA1c, mean (SD)	8.9 (1.8)
Use of metformin, n (%)	17 (77.2)

On the other hand, when patients were on thrice daily insulin injections, they had better average blood glucose readings compared to the twice daily regimen (161.4 ± 62.7and 166.0 ± 69.5 respectively, p < 0.05).

There was no significant difference in hypoglycemia incidents between the two regimens.

### Discussion

This trial is the first up to our knowledge to examine the efficacy and safety of BHI 30 three times a day when compared to BHI 30 two times daily. The idea of the trial came about while observing the general practice of physicians treating diabetics in Jordan. This practice is not based on any case-controlled studies. The medication is cheap and readily available in all health institutions. Long-standing Type 2 diabetics require high doses of insulin and the practice of giving BHI 30 thrice daily is thought of as a means of intensifying glucose control when use of insulin analogs is not feasible.

This study had shown that BHI 30 thrice daily was as effective and safe as BHI 30 twice daily.

Another interesting finding that might support this practice was that average glucose readings were significantly lower in thrice daily dosing regimen, although not reflected by HbA1c.

HbA1c is considered a gold-standard tool for monitoring chronic hyperglycemia, and a high correlation was found between HbA1c levels and mean blood glucose [7–9].

Shanmugasundar G et al. experimented with BHI 30 in an open-labelled randomized pilot study whereby 50 patients with uncontrolled T2DM on twice daily BHI and insulin sensitizers were randomized to either to BHI thrice daily or Basal-Bolus regimen. They came to the conclusion that a thrice daily BHI regimen is non-inferior to the basal-bolus insulin analogue regimen in terms of efficacy and safety in patients with poorly controlled T2DM [6].

One study that included 125 patients with type 1 diabetes mellitus were followed prospectively and randomized into trial and control groups. The control group received conventional two insulin injections per day: a mixture of short-acting (regular) + intermediated acting (NPH) insulins pre-breakfast (twice daily), and the trial group was treated by an extra dose of regular insulin before lunch (three times daily).

There was a significant decrease in HbA1c level in both groups (p < 0.05), but there was no significant difference in HbA1c reduction in patients on twice daily insulin injections and those on thrice daily insulin injection groups (1.12 ± 2.12 and 0.98 ± 2.1% respectively, p > 0.05).

They attributed better glycemic control to educating patients, frequent outpatient follow-up visits, and blood glucose monitoring rather than more frequent dosing of insulin [10].

As for the new premixed insulin analogues, many studies have shown the non-inferiority in efficacy and safety of administrations of these analogues in a thrice daily regimen [4, 5, 11–13].

However, a comparison of these studies to ours is relatively inapplicable because of the difference in types of insulin used.

Our results show a significant decrease in HbA1c in both groups at the end of the first 3-month interval regardless of the insulin regimen used. It is probably attributed to the education received at the start of trial and the frequent follow-up visits and titration of insulin doses.

The positive impact of structured education on glucose control in the management of Type 2 diabetes has been repeatedly demonstrated in many studies [14].

### Conclusion

Compared to the traditional twice daily insulin regimen of BHI 30, a therapeutic regimen involving dividing the same amount of daily insulin over 3 injections before meals and titrating up the dose according to glucose readings was comparable in terms of efficacy and safety in patients with poorly controlled type 2 Diabetes.

## Limitations

A major limitation would be the number of patients recruited. The study was designed originally to include 50 patients to strengthen the power of the study. Poor compliance to a more intensive insulin regimen and to self- monitoring of blood glucose, along with failure to comply with follow up visits, prevented a larger sample size.

## Supplementary information

**Additional file 1.** Raw data sheet.

## Data Availability

Attached as an excel sheet in Additional file 1.

## Abbreviations

BHI: Biphasic human insulin
HbA1c: Hemoglobin A1c
HI: Human insulin
NPH: Protamine-crystallized human insulin
SMBG: Self-monitoring of blood glucose
BIAsp: Biphasic insulin Aspart
T2DM: Type 2 DM
